# Histopathological findings of renal tissue induced by oxidative stress due to different concentrations of fluoride

**DOI:** 10.18632/oncotarget.17365

**Published:** 2017-04-21

**Authors:** Qin Luo, Hengmin Cui, Huidan Deng, Ping Kuang, Huan Liu, Yujiao Lu, Jing Fang, Zhicai Zuo, Junliang Deng, Yinglun Li, Xun Wang, Ling Zhao

**Affiliations:** ^1^ College of Veterinary Medicine, Sichuan Agricultural University, Wenjiang, Chengdu, China; ^2^ Key Laboratory of Animal Diseases and Environmental Hazards of Sichuan Province, Sichuan Agriculture University, Wenjiang, Chengdu, China

**Keywords:** sodium fluoride, oxidative damage, lesion, dysfunction, kidney, Immunology and Microbiology Section, Immune response, Immunity

## Abstract

It has been reported that excessive intake of fluoride can induce renal lesions. However, its pathogenesis is still less understood. Therefore, this study was conducted to investigate oxidative damage and the relationships between the oxidative damage and renal lesions in fluoride-treated mice by using the methods of histopathology, biochemistry, flow cytometry and quantitative real-time polymerase chain reaction (qRT-PCR). A total of 240 ICR mice were randomly divided into four equal groups (sodium fluoride was given orally at the dose of 0, 12, 24 and 48 mg/kg body weight for 42 days, respectively). We found that fluoride in excess of 12 mg/kg induced renal oxidative damage, which was characterized by increasing the levels of reactive oxygen species (ROS) production and contents of malondialdehyde (MDA) and protein carbonyls (PC), and decreasing the abilities of anti-superoxide anion (ASA) and anti-hydroxyl radical (AHR), glutathione (GSH) content, as well as activities and mRNA expression levels of superoxide dismutase (SOD), catalase (CAT), glutathione reductase (GR) and glutathione peroxidase (GSH-Px). Concurrently, fluoride caused degeneration and necrosis of the tubular cells, renal tubular hyaline casts and glomeruli swelling, which were consistent with the alteration of renal function parameters including elevated contents of serum creatinine (Cr), serum uric acid (UA), blood urea nitrogen (BUN), and the activities of urinary N-acetyl-b-D-glucosaminidase (NAG), renal lactate dehydrogenase (LDH), and reduced activities of sodium-potassium adenosine triphosphatase (Na+/K+-ATPase) and acid phosphatase (ACP) in the kidney. The above-mentioned results showed that fluoride in excess of 12 mg/kg induced renal oxidative damage, which then caused renal lesions and dysfunctions. These findings also clearly demonstrated that oxidative damage is one of the mechanisms of fluoride-induced renal lesions and dysfunctions.

## INTRODUCTION

Fluoride is widespread in the environment [1]. Low levels of fluoride are essential for the development of tooth enamel and bones, and often used in the medicine as antibiotic, psychopharmacology, anesthetics [2, 3]. High levels of fluoride have been found in groundwater of more than twenty developed and developing countries, including China [4, 5]. It has been demonstrated that excessive ingestion of fluoride can results in structural and functional changes in teeth and bones, such as mottling of teeth or skeletal fluorosis [6–9]. And along with teeth or skeletal fluorosis, pathological changes have also been reported in soft tissues including thyroid [10], thymus [11], brain [12, 13], heart [14, 15], liver [16–18], spleen [19–22], gastro-intestinal tract [23, 24], cecal tonsil [25, 26], bursa of Fabricius [27] and reproductive organs [28]. As the primary organ concerned with excretion and retention of fluoride, kidney is quite sensitive to the toxicity of fluoride [29]. Earlier studies have shown that excessive intake of fluoride causes a series of metabolic abnormalities in the kidney [30, 31]. However, the exact mechanism of fluoride-induced renal damage remains unclear at present.

Physiological and pathologicalmetabolic processes in living tissues give rise to the formation of reactive oxygen species (ROS), which can interact with both lipid and protein [32]. ROS mainly acts on bio-membranous unsaturated fatty acids, and decreases the membrane fluidity and disrupts the membranal structure and function [33]. The ROS levels are modulated by a complex network of antioxidant defence systems, and assisted by repair systems [34]. The situation of serious imbalance between oxidant and antioxidant is referred to as oxidative damage [35]. In many diseases, tissue damage is often accompanied by an imbalance in the oxidant/antioxidant status [36]. Previous studies have shown that fluoride exposure can increase the lipid peroxidation levels, and reduce the activities of glutathione peroxidase (GSH-Px), catalase (CAT) and superoxide dismutase (SOD) in the rat kidney [37, 38]. Guan et al [30] has also found that the lipid peroxidation levels of kidney are markedly increased in rats treated with high levels of fluoride, and speculated that the oxidative damage and modification of membrane lipids may be implicated in the pathogenesis of fluorosis. However, the exact mechanism of fluoride-induced renal oxidative damage is unclear at present.

Recently, it has been recognized that the gene expression occupies an important position in the risk assessment of environmental substances [39]. However, previous researches mainly focus on the activity changes of antioxidant enzymes in animals under fluoride exposure. There are no systematic reports about the molecular mechanisms of fluoride-induced renal oxidative damage in human beings and animals so far.

Therefore, the present study was conducted to explore the molecular mechanisms of fluoride-induced renal oxidative damage and the relationships between the oxidative damage and renal lesions in mice by observing the histopathological lesions and functional changes [represented by serum creatinine (Cr), serum uric acid (UA) and blood urea nitrogen (BUN) contents as well as urinary N-acetyl-b-D-glucosaminidase (NAG), renal lactate dehydrogenase (LDH), sodium-potassium adenosine triphosphatase (Na^+^/K^+^-ATPase) and acid phosphatase (ACP) activities], and the alteration of oxidative damage parameters including the levels of ROS production, contents of malondialdehyde (MDA), protein carbonyls (PC) and glutathione (GSH), abilities of anti-superoxide anion (ASA) and anti-hydroxyl radical (AHR), activities of SOD, CAT, glutathione reductase (GR) and GSH-Px as well as the mRNA expression levels of copper zinc superoxide dismutase (CuZn-SOD), manganese superoxide dismutase (MnSOD), CAT, GR and GSH-Px in the kidney of mice using the methods of histopathology, biochemistry, flow cytometry and quantitative real-time polymerase chain reaction (qRT-PCR).

## RESULTS

### Clinical signs

**Figure 1:**
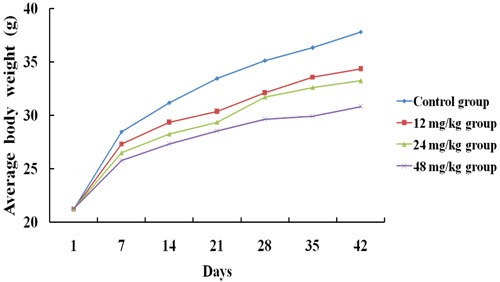
The average body weight of mice at 1, 7, 14, 21, 28, 35 and 42 days of the experimental

From 14 days of the experiment, the feed intake of mice in the 24 and 48 mg/kg groups began to decline in comparison with those of the control group. The mice in the 12, 24 and 48 mg/kg groups grew much slower than those in the control group (Figure 1). The fecal offensive odors of mice in the 24 and 48 mg/kg groups began in 35 days of the experiment. Meanwhile, a few of mice in the 48 mg/kg group showed polypnea and some neurologic symptoms such as rapid circular motion and extreme sensitivity to noise. No death was found during the experiment.

### Histopathological changes in the kidney

**Figure 2:**
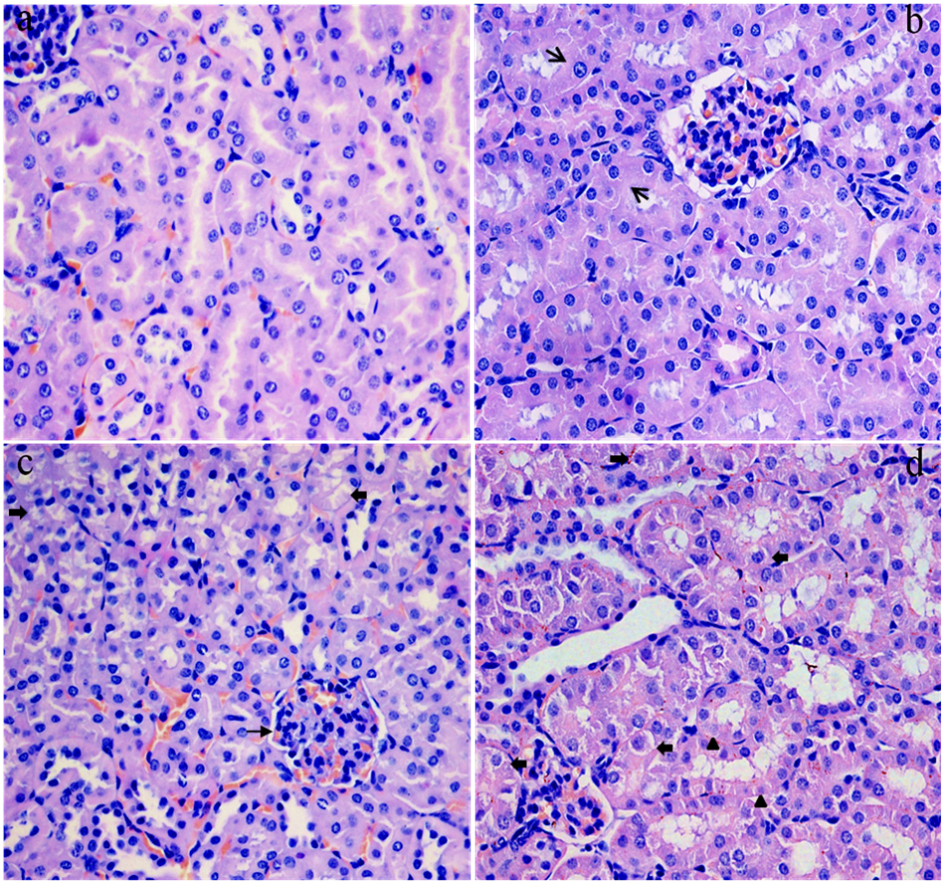
Histopathological changes in the kidney of mice at 21 days of the experiment (**a**) The control group. No changes are observed (H&E ×4 00). (**b**) The 12 mg/kg group. Tubular cells are swelled (↑, H&E × 400). (**c**) The 24 mg/kg group. Tubular cells show granular and vacuolar degeneration (↑). And the swelling glomeruli with narrow capsular space (↑) are observed (H&E × 400). (**d**) The 48 mg/kg group. Tubular cells show marked granularand vacuolar degeneration (↑). Also, few necrotic tubular cells (Δ) are observed (H&E × 400).

**Figure 3:**
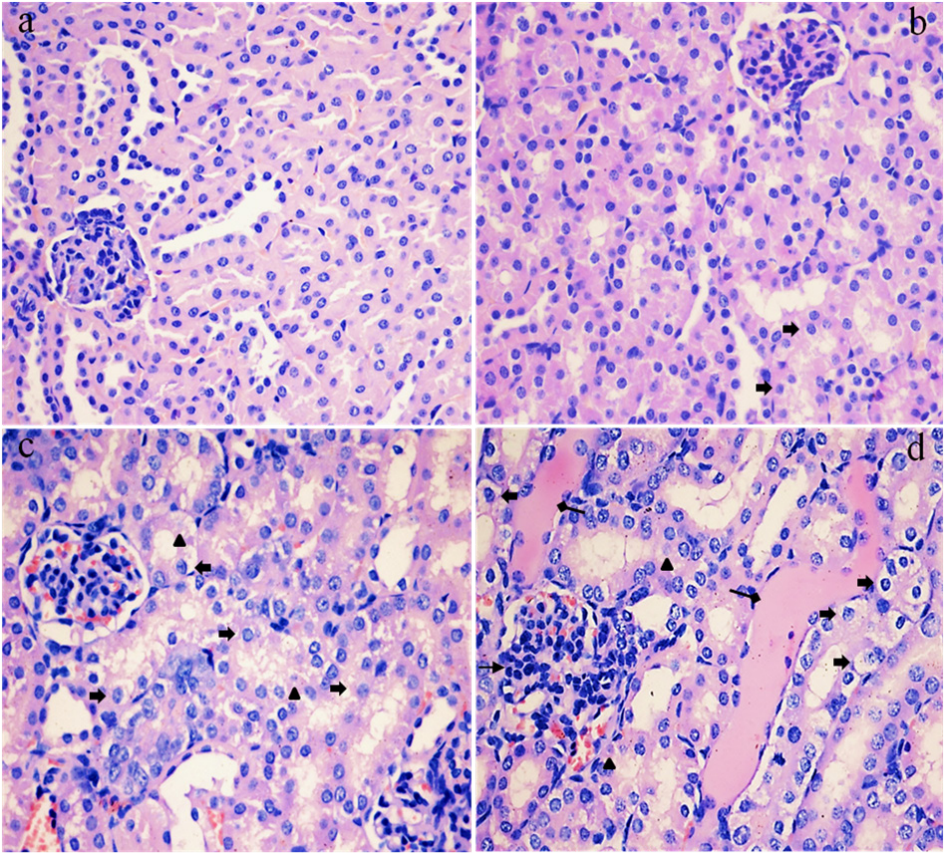
Histopathological changes in the kidney of mice at 42 days of the experiment (**a**) The control group. No changes are observed (H&E × 400). (**b**) The 12 mg/kg group. Tubular cells show slightly granular degeneration (↑, H&E × 400). (**c**) The 24 mg/kg group. Tubular cells show marked granularand vacuolar degeneration (↑). And some necrotic tubular cells (Δ) are observed (H&E × 400). (**d**) The 48 mg/kg group. Tubular cells show marked granularand vacuolar degeneration (Δ). Also, necrotic tubular cells (p), renal tubular hyaline casts (↑) and swelling glomeruli with a narrow capsular space (↑) are observed (H&E × 400).

As shown in Figures 2 and 3, the degeneration and necrosis of the tubular cells, glomeruli swelling as well as the renal tubular hyaline casts were observed in the experimental groups. Also, these histopathological lesions induced by fluoride were changed in a dose- and time-dependent manner. The above lesions were not observed in the control group.

### Changes of NAG activities in the urine

**Figure 4:**
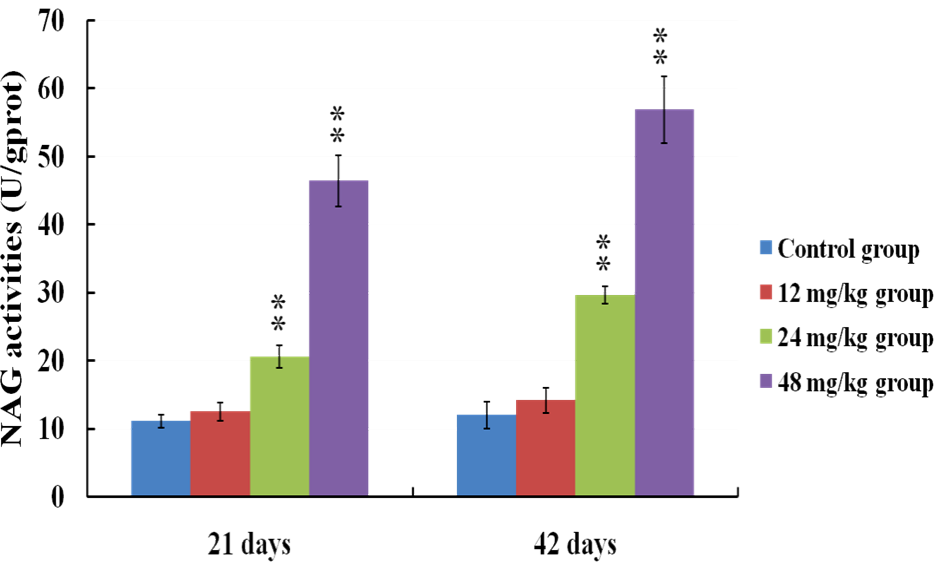
Changes of NAG activities in the urine at 21 and 42 days of the experiment Data are presented with the means ± standard deviation (*n* = 8). *p < 0.05, compared with the control group; **p < 0.01, compared with the control group.

Figure 4 showed that the activities of NAG were significantly increased (*p* < 0.01) in the 24 and 48 mg/kg groups at 21 and 42 days of the experiment when compared with those in the control group.

### Changes of BUN, Cr and UA contents in the serum

**Figure 5:**
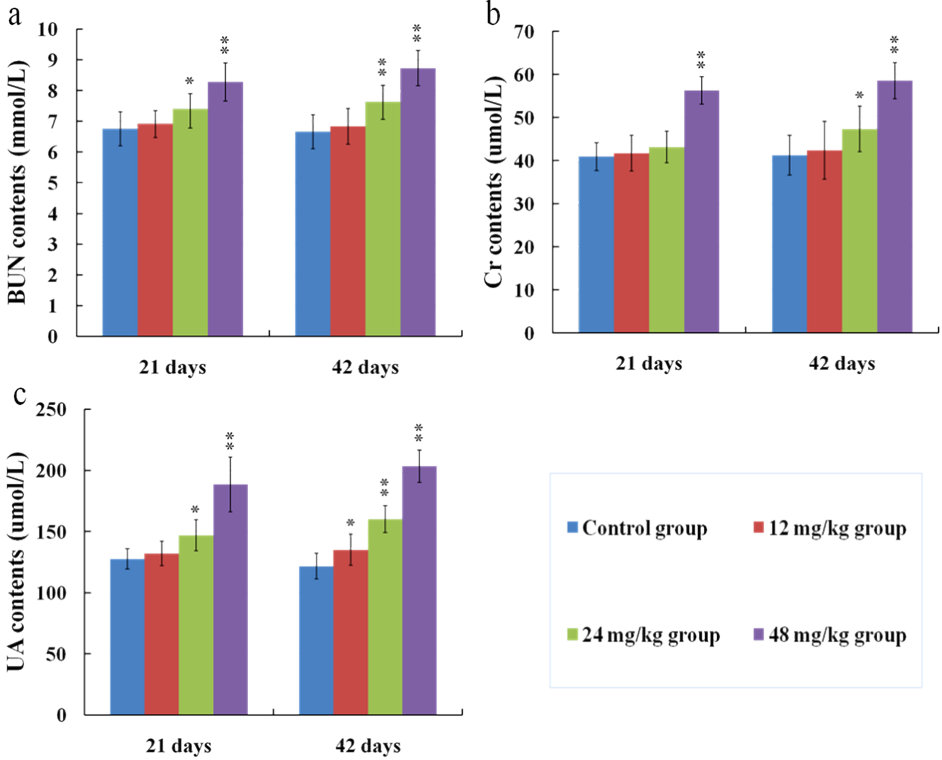
Changes of BUN (**a**), Cr (**b**) and UA (**c**) contents in the serum at 21 and 42 days of the experiment. Data are presented with the means ± standard deviation (*n* = 8). *p < 0.05, compared with the control group; **p < 0.01, compared with the control group.

The serum BUN (a), Cr (b) and UA (c) contents were markedly increased (*p* < 0.01 or *p* < 0.05) in the 24 and 48 mg/kg groups from 21 to 42 days of the experiment in comparison with those in the control group. The results were shown in Figure 5.

### Changes of Na^+^/K^+^-ATPase, ACP and LDH activities in the kidney

**Figure 6:**
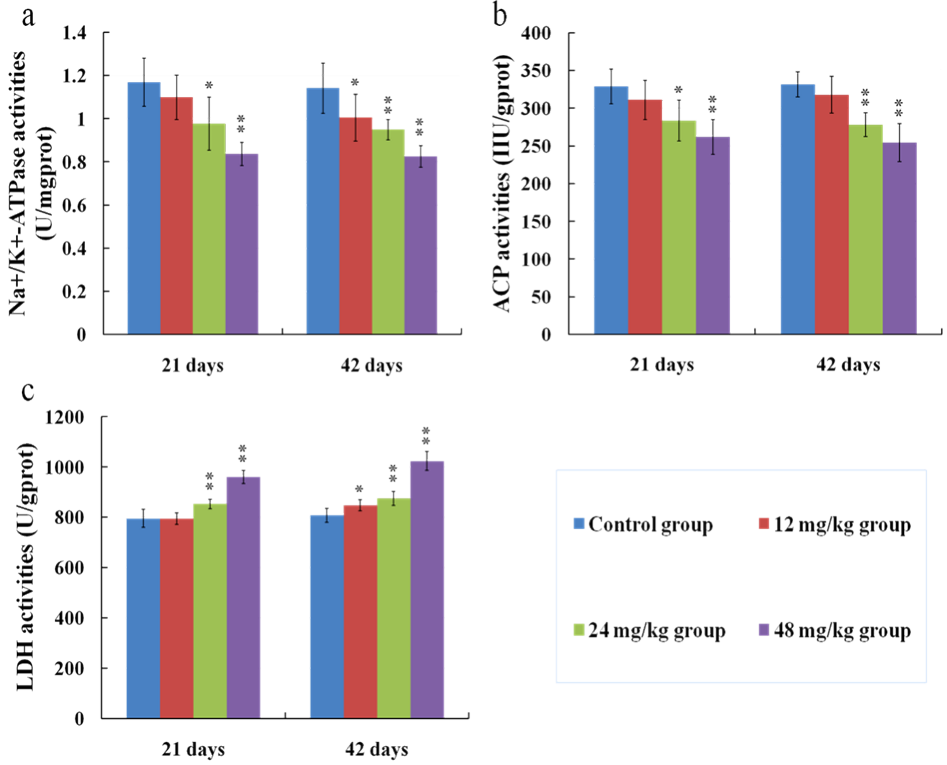
Changes of Na+/K+-ATPase (**a**), ACP (**b**) and LDH (**c**) activities in the kidney at 21 and 42 days of the experiment. Data are presented with the means ± standard deviation (*n* = 8). *p < 0.05, compared with the control group; **p < 0.01, compared with the control group.

The results in Figures 6a-6b showed that the Na^+^/K^+^-ATPase and ACP activities in the kidney were lower (*p* < 0.01 or *p* < 0.05) in the 24 and 48 mg/kg groups at 21 and 42 days than those in the control group. The LDH activities were increased (*p* < 0.05) in the 12 mg/kg group at 42 days of the experiment and were significantly increased (*p* < 0.01) in the 24 and 48 mg/kg groups at 21 and 42 days of the experiment when compared with those in the control group, as shown in Figure 6c.

### Changes of ROS production levels in the kidney

**Figure 7:**
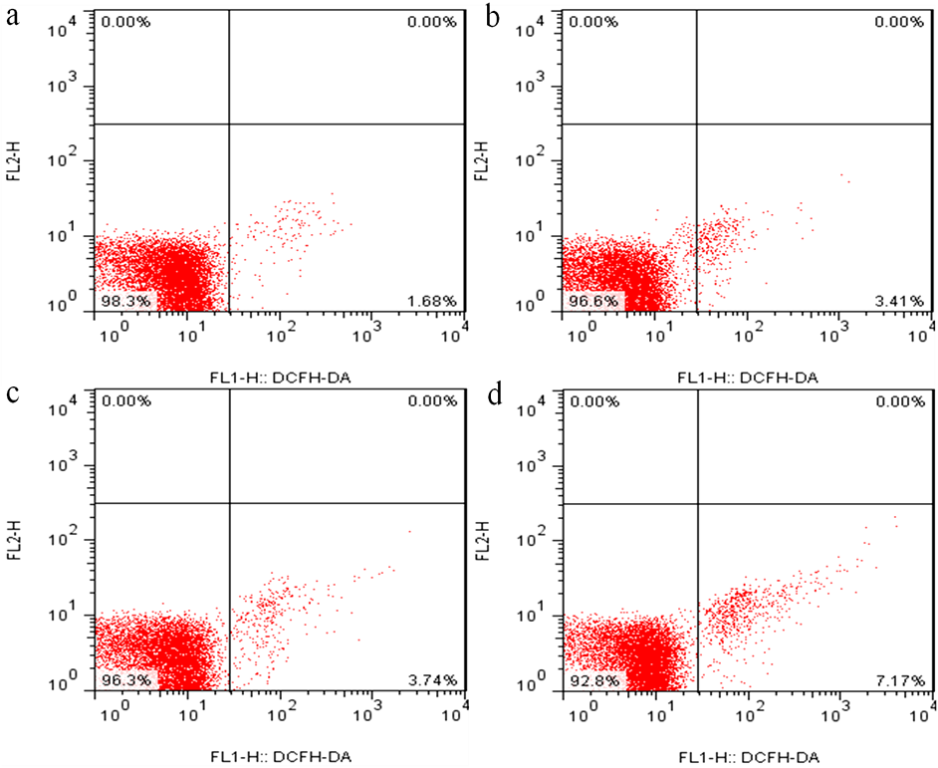
The diagram of renal ROS production levels obtained by flow cytometry at 21 days of the experiment

**Figure 8:**
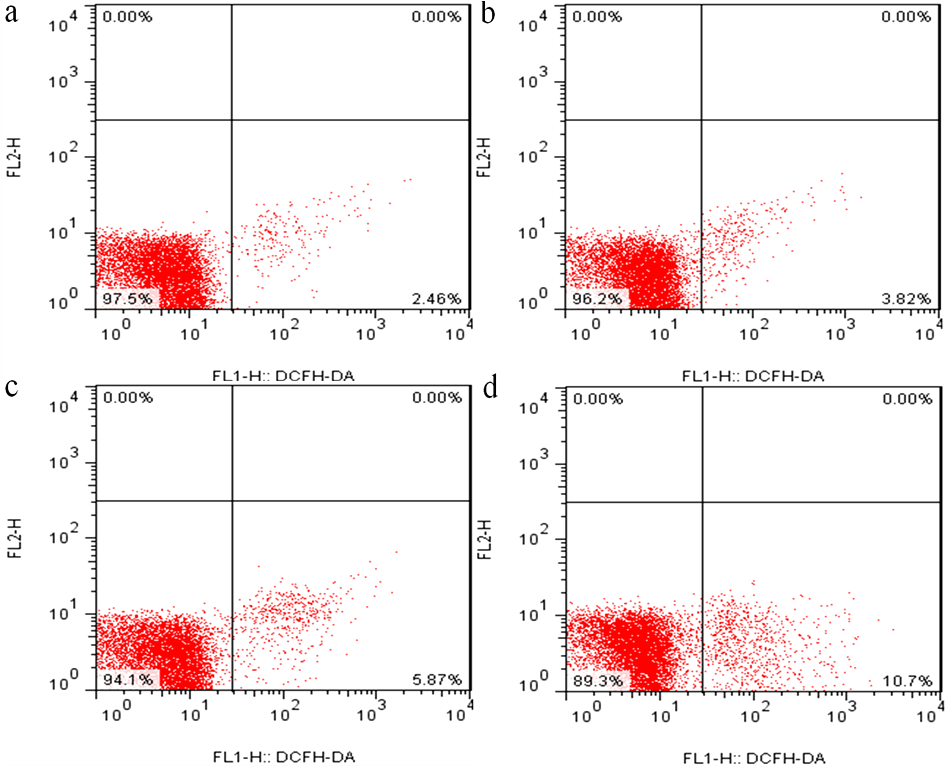
The diagram of renal ROS production levels obtained by flow cytometry at 42 days of the experiment

**Figure 9:**
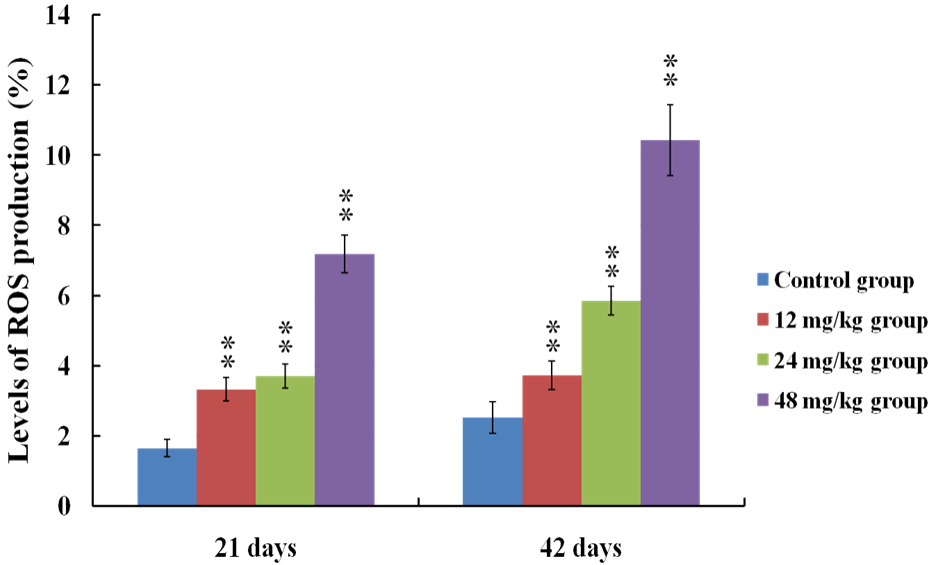
Changes of ROS production levels in the kidney at 21 and 42 days of the experiment Data are presented with the means ± standard deviation (*n* = 8). *p < 0.05, compared with the control group; **p < 0.01, compared with the control group.

Figures 7, 8 and 9 showed that the levels of ROS production were significantly enhanced (*p* < 0.01) in the 12, 24 and 48 mg/kg groups at 21 and 42 days of the experiment in comparison with those in the control group.

### Changes of MDA and PC contents in the kidney

**Figure 10:**
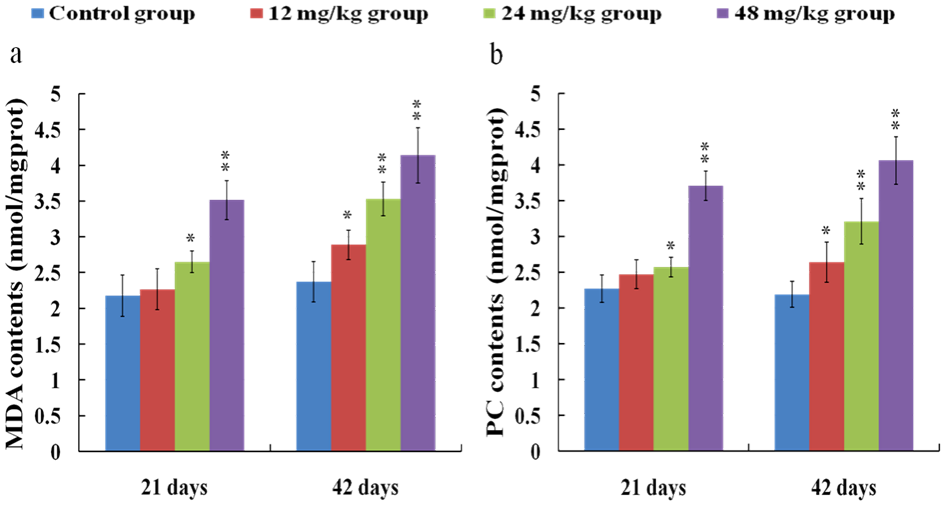
Changes of MDA (**a**) and PC (**b**) contents in the kidney at 21 and 42 days of the experiment. Data are presented with the means ± standard deviation (*n* = 8). *p < 0.05, compared with the control group; **p < 0.01, compared with the control group.

As shown in Figure 10, the MDA (a) and PC (b) contents were higher (*p* < 0.05) in the 12 mg/kg group at 42 days of the experiment and were markedly increased (*p* < 0.01 or *p* < 0.05) in the 24 and 48 mg/kg groups at 21 and 42 days of the experiment than those in the control group.

### Changes of ASA and AHR abilities in the kidney

**Figure 11:**
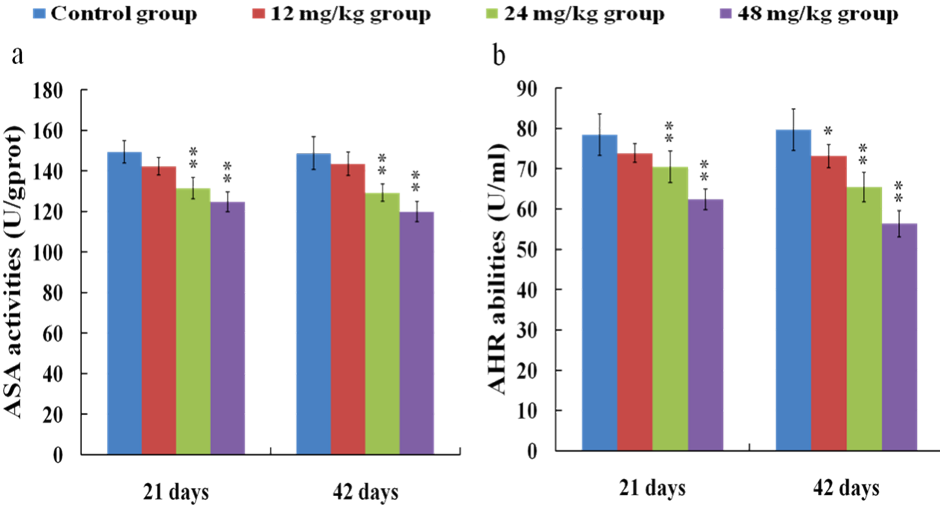
Changes of ASA (**a**) and AHR (**b**) abilities in the kidney at 21 and 42 days of the experiment. Data are presented with the means ± standard deviation (*n* = 8). *p < 0.05, compared with the control group; **p < 0.01, compared with the control group.

ASA (a) and AHR (b) abilities were significantly decreased (*p* < 0.01) in the 24 and 48 mg/kg groups at 21 and 42 days of the experiment in comparison with those in the control group. The results were shown in Figure 11.

### Changes of antioxidant enzyme activities and GSH contents in the kidney

**Figure 12:**
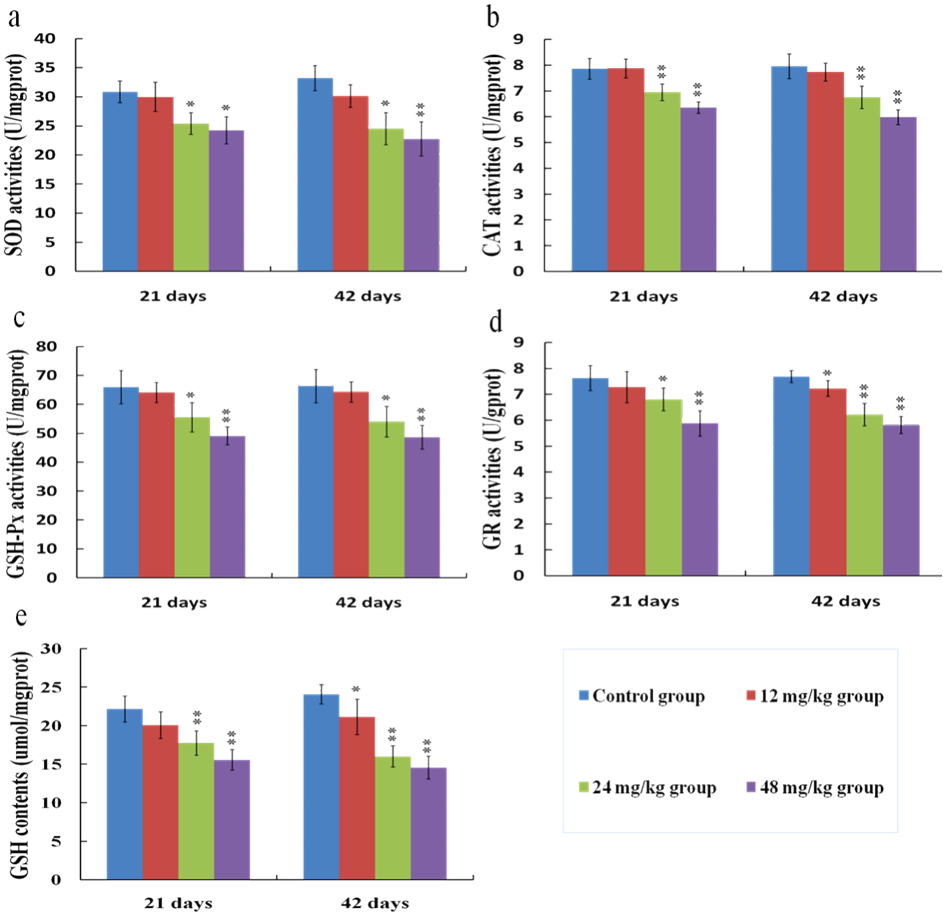
Changes of SOD (**a**), CAT (**b**), GSH-Px (**c**), GR (**d**) activities, and GSH (**e**) contents in the kidney at 21 and 42 days of the experiment. Data are presented with the means ± standard deviation (*n* = 8). *p < 0.05, compared with the control group; **p < 0.01, compared with the control group.

SOD (a), CAT (b), GSH-Px (c) and GR (d) activities, and GSH contents (e) were reduced (*p* < 0.05) in the 24 and 48 mg/kg groups at 21 and 42 days of the experiment when compared with those in the control group, as shown in Figure 12.

### Changes of antioxidant enzyme mRNA expression levels in the kidney

**Figure 13:**
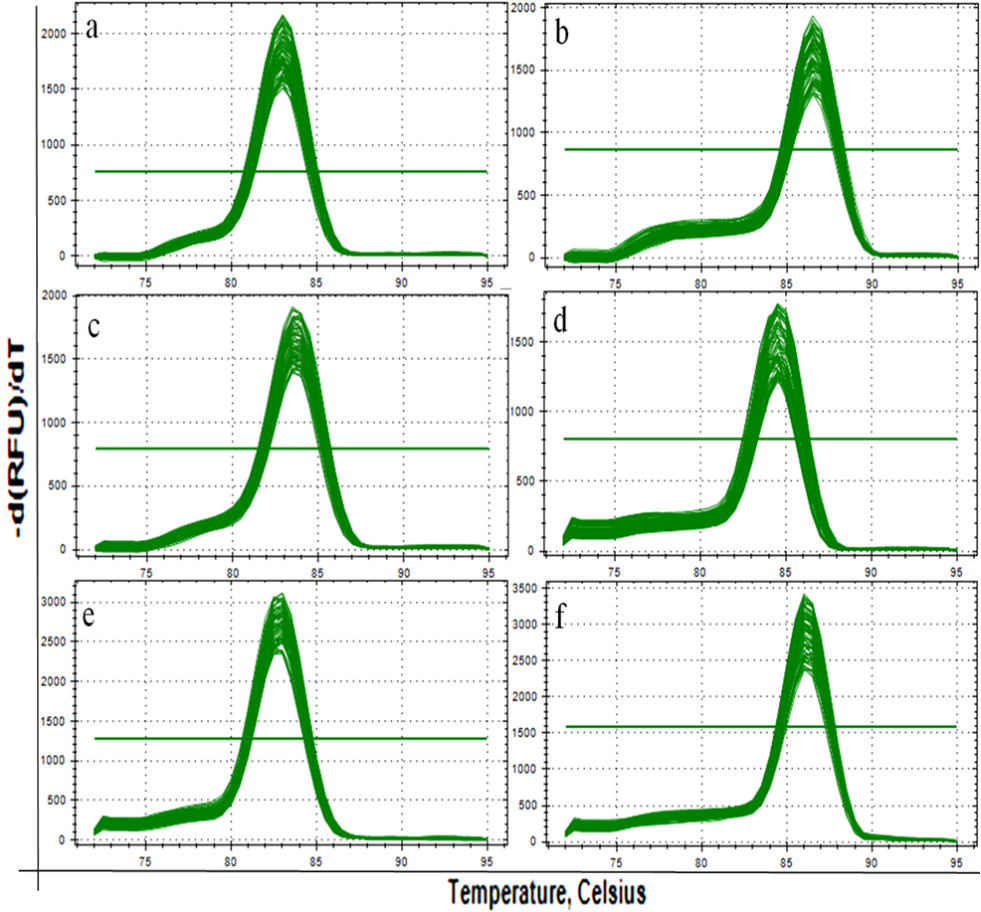
The melting curve analysis of CuZn-SOD (**a**), MnSOD (**b**), CAT (**c**), GSH-Px (**d**) and GR (**e**) in the kidney. There is only one peak for each PCR product.

**Figure 14:**
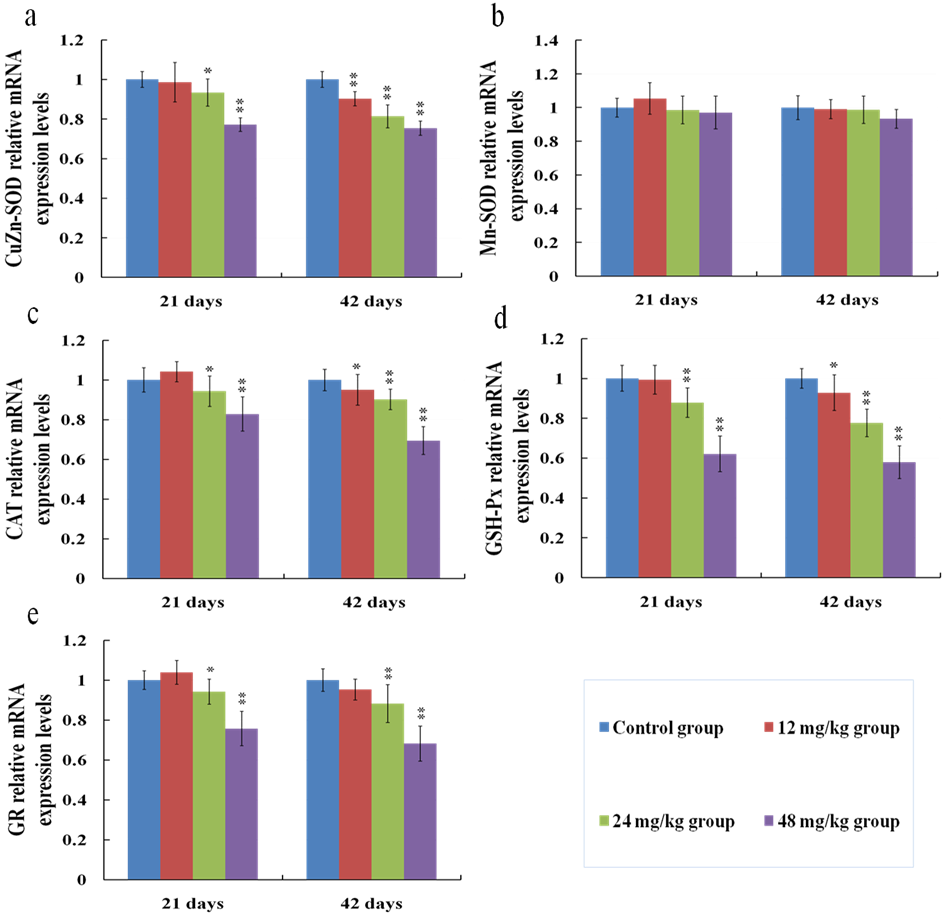
Changes of mRNA expression levels of CuZn-SOD (**a**), MnSOD (**b**), CAT (**c**), GSH-Px (**d**) and GR (**e**) in the kidney at 21 and 42 days of the experiment. Data are presented with the means ± standard deviation (*n* = 8). *p < 0.05, compared with the control group; **p < 0.01, compared with the control group.

The melting curve analysis of CuZn-SOD (a), MnSOD (b), CAT (c), GSH-Px (d) and GR (e) were showed in Figure 13, and there was only one peak for each PCR product. Meanwhile, the mRNA expression levels of CuZn-SOD (a), CAT (c), GSH-Px (d) and GR (e) were decreased (*p* < 0.01 or *p* < 0.05) in the 12, 24 and 48 mg/kg groups in comparison with those in the control group at 21 and 42 days of the experiment, while the mRNA expression levels of MnSOD (b) were not significantly changed between the fluoride exposure groups and the control group, as shown in Figure 14.

## DISCUSSION

The present study focuses on the effects of fluoride on kidney because kidney is crucial for the regulation of body homeostasis and excretion of metabolic wastes [40]. To our knowledge, this is the first report to systematic investigate the renal oxidative damage induced by fluoride and the relationships between the oxidative damage and renal lesions in human beings and animals at present.

To establish the animal model of renal damage induced by fluoride, the experimental mice were treated with graded levels of fluoride (0, 12, 24 and 48 mg/kg, respectively) for 42 days. The dose of fluoride used in this study was based on the LD50 value (median lethal dose value) of the acute oral toxicity study and the environmentally realistic concentrations of fluoride [41]. And our results showed that the histopathological lesions of kidney such as degeneration and necrosis of the tubular cells, glomeruli swelling as well as the renal tubular hyaline casts were changed at a dose- and time-dependent manner from 21 to 42 days during the experiment. According to the close relationship between structure and function, the renal function will be reduced when the morphological structure of renal tubules and glomeruli are destroyed [42]. In the present study, the contents of serum Cr, UA and BUN as well as the activities of urinary NAG and renal LDH were increased, while the activities of renal Na^+^/K^+^-ATPase and ACP were decreased in the 12, 24 and 48 mg/kg groups, demonstrating that the renal function was reduced or impaired by fluoride. And our results are consistent with the report that fluoride can increase the NAG activities in urine of children [43].

The renal histopathology lesions and function parameters are gradually changed in a dose- and time-dependent manner, suggesting that the fluoride is main and/or direct reason for the renal damage in this study. However, the detailed effects of fluoride-induced oxidative damage on the mechanisms of renal lesions are not very clear today.

It is well known that the increased ROS production is involved in the toxic effects of a variety of compounds and the pathogenesis of many diseases [24, 44]. Although the ROS production also has been considered as an important cause in fluoride-caused toxicity, the ROS production in the kidney of mice induced by fluoride is not very clear [45, 46]. Therefore, we firstly measured the renal ROS production in mice, and found that the levels of ROS production were significantly enhanced in the 12, 24 and 48 mg/kg groups, suggesting that fluoride exposure induced the ROS production. Excessive ROS production can oxidize cell components such as lipid and protein, leading to lipid peroxidation and protein oxidation [47]. And MDA and PC are the representative products of lipid peroxidation and protein oxidation, respectively [48]. Thus, we next investigated the MDA and PC contents in the kidney. The results showed that the MDA and PC contents were increased in the 12, 24 and 48 mg/kg groups, indicating that fluoride exposure caused lipid and protein oxidative damage in the kidney, which was attributed to the increase of ROS production. A balance between the production and elimination of ROS such as superoxide anion and hydroxyl radical regulates the cellular redox homeostasis [49]. ASA and AHR abilities were often used to assess the total capacity of scavenging superoxide anion and hydroxyl radical, respectively [50]. In this study, the ASA and AHR abilities were decreased in the 24 and 48 mg/kg groups when compared with those in the control group, which suggested that the renal ROS scavenging capacity was reduced, and the balance between the production and elimination of ROS was disrupted by fluoride. The ROS scavenging system of living organisms mainly consist of enzymatic and non-enzymatic antioxidants [51]. Therefore, in order to study the pathways of fluoride-reduced ROS scavenging capacity, we next observed the effects of fluoride on the activities of antioxidant enzymes and contents of non-enzymatic antioxidant in the kidney.

Free radical-scavenging enzymes such as SOD and CAT are identified as the first line of cellular defense against oxidative damage [52]. SOD is the primary enzyme to respond against the superoxide radicals, and converts the superoxide radicals into hydrogen peroxide [53]. CAT is responsible for the breakdown of hydrogen peroxide and protects the tissues from the potential toxicity of hydroxyl radicals [51]. Consequently, the effects of SOD and CAT are complementary to each other [54]. In this study, it was found that the SOD and CAT activities were decreased in the 24 and 48 mg/kg groups, suggesting that the superoxide radicals and/or hydroxyl radicals were accumulated in the kidney. GSH can scavenge various reactive species through a non-enzymatic mechanism, and the perturbation in the redox status of GSH will enhance oxidative stress and tissue damage [36]. Meanwhile, GSH-dependent enzymes such as GSH-Px and GR are able to counteract the oxidative damage [40]. GSH-Px works together with CAT to scavenge excess of lipid peroxide and hydrogen peroxide [53]. But unlike CAT, GSH-Px removes hydrogen peroxide by oxidizing GSH to oxidized glutathione (GSSG) [55]. GR, a flavoprotein enzyme, converts GSSG back into GSH by using the reducing power of NADPH [56]. In the present study, the GSH contents and GR and GSH-Px activities were reduced by fluoride, which were consistent with the results obtained by Chen [57]. And the reduced GR and GSH-Px activities may be related to the accumulation of free radicals and/or the depletion of GSH induced by fluoride.

In this study, the decreased SOD, CAT, GR and GSH-Px activities as well as the GSH contents indicated that fluoride depleted enzymatic and non-enzymatic antioxidants. To show the molecular basis of the changes of antioxidant enzyme activities, we investigated the mRNA expression levels of antioxidant enzymes in the kidney.

The results in the present study showed that fluoride exposure decreased the mRNA expression levels of CuZn-SOD, CAT, GR and GSH-Px, which were consistent with the reduction of these antioxidant enzyme activities. Additionally, MnSOD mRNA expression levels were not changed. It might be partly because MnSOD is distributed in the mitochondrion [58], which mainly scavenges the excessive radicals that come from mitochondrial respiration, whereas fluoride might mainly be involved in assaults via the cell membrane.

## CONCLUSIONS

**Figure 15:**
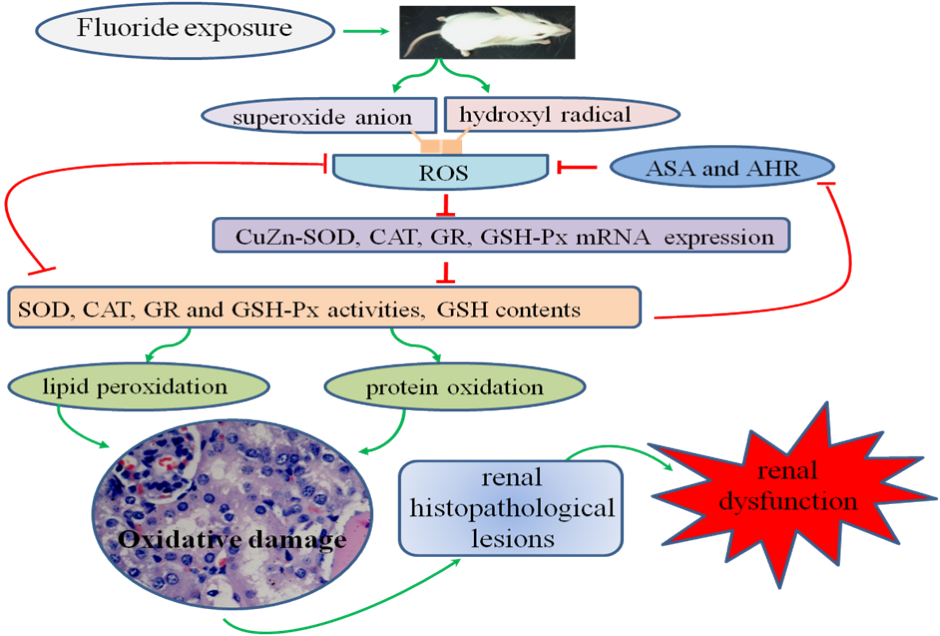
Schematic of fluoride-induced renal oxidative damage as well as the relationships between the oxidative damage and renal lesions in mice treated with graded levels of fluoride Fluoride can enhance the renal oxidative damage by increasing the ROS production and contents of MDA and PC, and reducing the free radical-scavenging ability as well as the mRNA expression levels and activities of antioxidant enzymes, which then cause renal lesions and dysfunctions. Oxidative damage is one of the mechanisms of fluoride-induced renal lesions and dysfunctions.

Results in this study showed that fluoride in excess of 12 mg/kg can enhance the renal oxidative damage by increasing the ROS production and MDA and PC contents, and reducing the free radical-scavenging ability, and the mRNA expression levels and activities of antioxidant enzymes, which then cause renal lesions and dysfunctions. This study also clearly demonstrated that oxidative damage is one of the pathways of fluoride-induced renal lesions and dysfunctions, as shown in Figure 15.

## MATERIALS AND METHODS

### Chemicals

Sodium fluoride was purchased from Chengdu Kelong Chemical Co., Ltd. (Chengdu, China). ROS Assay kit (S0033) was obtained from Beyotime Biotechnology, China. Reagent kits for determination of biochemical parameters were purchased from Nanjing Jiancheng Bioengineering Institute of China (Nanjing, China). RNAiso Plus, Prim-Script^™^ RT reagent Kit and SYBR^®^ Premix Ex Taq^™^ II were purchased from Takara Biotechnology (Dalian) Co., Ltd. (Dalian, Liaoning, China). All other chemicals used in the experiment were analytical grade.

### Experimental animals and treatment

A total of 240 four-week-old ICR mice obtained from the Chengdu Dossy Experimental Animals Co., Ltd. [License No. SCXK (Sichuan) 2008-24] were used for the present study. The animals were housed in separate polypropylene cages, and kept under the standard laboratory conditions (at a temperature of 20-22 °C with a 12 h light/12 h dark photoperiod and 55-60% humidity). Diet and water were provided *ad libitum* throughout the experimental period.

After one week of acclimation, animals were randomly divided into four equal groups with 60 mice in each. Group I was given orally distilled water only and served as untreated (control) group. Groups II, III and IV were given orally fluoride (in the form of sodium fluoride) at the dose of 12, 24 and 48 mg/kg body weight, respectively. Mice were administered their respective doses daily by gavages for consecutively 42 days, and the gavage volume was 1ml/100g body weight.

All experimental procedures involving the use of mice were approved by the Animal Care and Use Committee, Sichuan Agricultural University.

### Clinical and histopathological observation

During the course of experiment, clinical signs and body weight gain of all animals were observed and recorded periodically. At 21 and 42 days of the experiment, eight mice in each group were humanly sacrificed for histopathological observation. Kidneys were immediately removed, fixed in 4% paraformaldehyde, dehydrated with increasing concentrations of ethanol, cleared with xylene and embedded in paraffin. And then kidneys were serial sectioned at 5 μm thickness, stained with hematoxylin and eosin, and observed by light microscopy.

### Determination of NAG activities in the urine

At 20 and 41 days of the experiment, eight mice in each group were placed into special metabolic cages for collecting 24 hour urine samples. The activities of NAG in the urine samples were detected by biochemical methods following the instructions of the reagent kit (NAG, A031; Nanjing Jiancheng, China).

### Determination of BUN, Cr and UA contents in the serum

At 21 and 42 days of the experiment, blood samples were collected from retro-ocular artery of every eight mice in each group without anticoagulant. Serum was separated by centrifugation at 3000 rpm for 15 min. Then the contents of BUN, Cr and UA in serum were detected by biochemical methods following the instructions of the reagent kits (BUN, C013-2; Cr, C011-2; UA, C012-2; Nanjing Jiancheng, China).

### Determination of Na^+^/K^+^-ATPase, ACP and LDH activities in the kidney

After blood samples collection, the same mice were sacrificed and kidneys were removed immediately. Kidneys were washed using chilled saline solution, weighed, homogenized in nine volumes of ice-cold 0.9% NaCl solution and centrifuged at 3500 rpm for 10 min at 4°C. Then the supernatant was collected for the determination of Na^+^/K^+^-ATPase, ACP and LDH activities by biochemical methods following the instructions of the reagent kits (Na^+^/K^+^-ATPase, A016-1; ACP, A060-2; LDH, A020-2; Nanjing Jiancheng, China).

### Determination of ROS production in the kidney by flow cytometry

At 21 and 42 days of the experiment, kidneys of every eight mice in each group were taken to measure the levels of ROS production by flow cytometry. Kidneys were crushed, filtered with 350 mesh nylon membrane, centrifuged (600 × g, 5min), and adjusted to a cell density of 1.0×10^6^ cells/ml with phosphate-buffered saline (PBS). 300 μL cell suspensions were taken and transferred to another centrifuge tube, and stained with 10μM DCFH-DA for 20 min at 37°C. Then the cells were washed with PBS and centrifuged (600 × g, 5min) once more. The supernatant was discarded, and cells were resuspended in 0.5 ml PBS and counted by BD FACS Calibur flow cytometer within 45 min.

### Determination of the oxidative and anti-oxidative parameters in the kidney

At 21 and 42 days of the experiment, eight mice in each group were sacrificed and kidneys were removed quickly. Then kidneys were homogenized in nine volumes of ice-cold 0.9% NaCl solution, and supernatant was collected for the following determination of oxidative stress parameters.

The total protein, MDA (a marker of lipid peroxidation), PC (a marker of proteinoxidation) and GSH contents, ASA and AHR abilities, and SOD, CAT, GR, GSH-Px activities in the kidney were determined by biochemical methods according to the instructions of the reagent kits (total protein, A045-3; MDA, A003-1; PC, A087; GSH, A006-2; ASA, A052; AHR, A018; SOD, A001-1; CAT, A007-1; GR, A062; GSH-Px, A005; Nanjing Jiancheng, China).

### Determination of antioxidant enzyme mRNA expression levels in the kidney by qRT-PCR

At 21 and 42 days of the experiment, kidneys of eight mice in each group were removed, stored in liquid nitrogen, and then homogenized with liquid nitrogen for RNA extraction. The methods of RNA extraction and qRT-PCR analysis were same as the described by Guo et al [59]. Briefly, the total RNA of the kidneys were extracted using RNAiso Plus (9109; Takara, China) following the manufacturer's instructions. The cDNA, used as the template for qRT-PCR analysis, was synthesized using a Prim-Script^TM^ RT reagent Kit (RR047A, Takara, China) following the manufacturer's instructions. Specific primers for the genes were designed and synthesized by Sangon (Shanghai, China) according to the Mus musculus sequences, and the expression levels of these genes were normalized to the expression levels of a housekeeping gene, β-actin (Table 1).

qRT-PCR was performed on a Thermal Cycler (C1000, BIO RAD, USA) using SYBR® Premix Ex Taq^TM^ II (DRR820A, Takara, China) according to the standard protocols. Gene expression values of the control group at 21 and 42 days of the experiment were used for gene expression calibration. The results of qRT-PCR were analyzed by 2^−ΔΔCT^method [60].

**Table 1:** Primer sequences of genes selected for analysis by qRT-PCR

Target gene	Accession number	Primer	Primer sequence (5′-3′)	Product size	Tm (°C)
CuZnSOD	NM_011434	Forward	GGACAATACACAAGGCTGTACC	113bp	61
		Reverse	CAGTCACATTGCCCAGGTCTC		
Mn-SOD	NM_013671	Forward	CAGACCTGCCTTACGACTATGG	113bp	61
		Reverse	CTCGGTGGCGTTGAGATTGTT		
GSH-Px	NM_008160	Forward	TACACCGAGATGAACGATCTG	102bp	57
		Reverse	ATTCTTGCCATTCTCCTGGT		
CAT	NM_009804	Forward	CCTATTGCCGTTCGATTCTC	119bp	61
		Reverse	CCCACAAGATCCCAGTTACC		
GR	NM_010344	Forward	AAGCGCTTCTCACCCCAGTT	121bp	61
		Reverse	GGGTGGCTGAAGACCACAGTA		
β-actin	NM_007393	Forward	GCTGTGCTATGTTGCTCTAG	117bp	59
		Reverse	CGCTCGTTGCCAATAGTG		

### Statistical analysis

The SPSS 17.0 statistical software package programme for Windows was used for statistical tests. All results were expressed as mean ± standard deviation. Differences between group means were estimated using one way analysis of variance (ANOVA). A value of *p* < 0.05 or *p* < 0.01 was accepted as statistically significant differences.
